# The Anti-Fatigue Effect of Glycoprotein from Hairtail Fish (*Trichiurus lepturus*) on BALB/c Mice

**DOI:** 10.3390/foods12061245

**Published:** 2023-03-14

**Authors:** Xiaodan Lu, Jiaqi Chen, Luyao Huang, Yujia Ou, Jingru Wu, Zebin Guo, Baodong Zheng

**Affiliations:** 1 Engineering Research Centre of Fujian-Taiwan Special Marine Food Processing and Nutrition, Ministry of Education, Fuzhou 350002, China; 2 College of Food Science, Fujian Agriculture and Forestry University, Fuzhou 350002, China

**Keywords:** anti-fatigue, antioxidant, hairtail fish glycoprotein

## Abstract

Fatigue is related to a variety of chronic diseases and has become a hot research topic in recent years. Various bioactive components have been extracted from hairtail fish (*Trichiurus lepturus*); however, none of these studies involved the anti-fatigue activity of hairtail fish glycoprotein (HGP). Thus, antioxidant experiments were conducted in vitro, and the anti-fatigue activity of HGP was further evaluated in BALB/c mice. The effects of HGP on the behavior of BALB/c mice were verified by classical behavioral experiments, and the indicators related to anti-fatigue activity were detected. The results showed that the antioxidant capacity in vitro of HGP increased gradually in the concentration range of 10 to 100 mg/mL. HGP improved the exercise ability of the mice. HGP was also found to significantly (*p* < 0.05) reduce the serum levels of lactate dehydrogenase (LDH), blood lactic acid (BLA), blood urea nitrogen (BUN), and creatine kinase (CK). The contents of liver glycogen (LG) and muscle glycogen (MG) were also significantly (*p* < 0.05) increased by HGP. Malondialdehyde (MDA) content in the serum and brains of the mice was significantly (*p* < 0.05) reduced and catalase (CAT), glutathione peroxidase (GPX), and superoxide dismutase (SOD) were significantly (*p* < 0.05) increased by HGP, especially in the middle- and high-dose groups. These results enhance our understanding of the anti-fatigue function of HGP and lay an important foundation for the further development and utilization of hairtail fish resources.

## 1. Introduction

The rapid pace of modern life means that people are often in a state of psychological stress and tension and suffer from a lack of sleep and fatigue [1,2]. Fatigue can cause memory decline, low work efficiency, lethargy and lack of energy, and even some diseases [3,4,5]. The health and safety risks associated with fatigue have received extensive attention [6,7]. Fatigue is mainly caused by excessive mental or physical activity, which is explained by the accumulation of free radicals [8], exhaustion of energy substances [9], excessive accumulation of metabolites [10], and oxidative stress [11]. Especially in the process of long-term exercise, the energy consumption of cells increases, ATP synthesis decreases, and the adjustment ability of the body decreases, which leads to a state of exercise-induced fatigue [12,13,14]. The relief and recovery of fatigue have become a research hotspot at home and abroad. Current fatigue relief methods include sleep supplements, drug relief, and nutritional supplementation [15]. Due to growing health concerns, there are many anti-fatigue products on the market; however, there are health concerns around their often subtle side effects.

In recent years, the application of natural products in nutritional intervention for fatigue has attracted increased attention [1]. Anti-fatigue active substances such as polypeptides [16], polysaccharides [17,18], and proteins [19] are characterized by high safety, no side effects, and significant efficacy. Therefore, using natural active ingredients to develop natural functional products has become an urgent need in the market. Glycoproteins are bioactive binding proteins composed of sugar and polypeptide chains connected by covalent bonds. They play an important role in protein folding, information transmission, nerve conduction, and molecular recognition [20,21]. Some studies have found that glycoproteins have a variety of functional activities, such as antioxidant, anti-tumor, and anti-inflammatory [22,23,24], and they have broad application prospects in the development of functional foods and new drugs. Some studies have confirmed that many foodborne glycoproteins have antioxidant [25], anti-fatigue [26], and other functions.

Marine resources have potential economic value and are important in the alleviation of the global energy shortage [27]. China accounts for >60% of global aquaculture production [28]. In recent years, the aquatic processing industry has developed rapidly. Hairtail fish (*Trichiurus lepturus*) are rich in nutrients [29] and are an excellent source of bioactive substances [30], such as glycoproteins. Marine fish bioactive substances have good safety and no side effects and are good supplements for fatigue. Therefore, many researchers have studied the anti-fatigue activity of marine natural bioactive components [15,16,31,32,33]. However, there are no relevant studies on the anti-fatigue activity of hairtail fish glycoprotein (HGP).

In this study, we conducted antioxidant experiments in vitro, and the anti-fatigue activity of HGP was further evaluated in an animal model. Our results provide a theoretical basis for the high-value utilization of hairtail fish and for research on the anti-fatigue mechanism of HGP and the development of functional foods.

## 2. Materials and Methods

### 2.1. Materials

Hairtail fish without guts were provided by Hai Xin Foods Co., Ltd. (Fuzhou China) and stored at −20 °C. BALB/c mice were purchased from Wu’s Laboratory Animal Co., Ltd. (Fuzhou, China). All kits were purchased from Solarbio Technology Co., Ltd. (Beijing, China). Other reagents were analytical pure reagents.

### 2.2. Preparation of HGP

The frozen hairtail fish without guts were thawed naturally, after which NaCl solution (0.4 mol/L) was added [34] with a solid–liquid ratio of 1:9 for ultrasonic crushing (525 W, 20 min) (XH-300B, Beijing, China) and then extracted in a water bath for 5 h at 60 °C and centrifuged (4 °C, 11,190× *g*, 10 min). The supernatant was added to ethanol until the final concentration reached 75% (*v*/*v*). After the precipitate was taken and dissolved, the protein was removed by adding Sevage reagent (chloroform and n-butanol, 4:1) with a volume ratio of 1:3. The supernatant was removed after centrifugation (4 °C, 11,190× *g*, 10 min). The collected precipitate was transferred to a dialysis bag. The appropriate permeation and cutoff membranes were selected for separation experiments (0.3–0.4 MPa, 25–45 °C) after dialysis for >48 h. The concentrated solution was collected and freeze-dried to obtain HGP. The successful separation of HGP was confirmed by sodium dodecyl sulfate poly acrylamide gel electrophoresis (SDS-PAGE).

### 2.3. Antioxidant Activity of HGP

The standard curve of total antioxidant capacity was obtained with FeSO_4_·7H_2_O as the standard. Total antioxidant capacity and the scavenging rates of hydroxyl radical, superoxide anion, DPPH radical, and ABTS radical were determined according to the kit instructions. All kits were purchased from Beijing Solarbio Science & Technology Co., Ltd. (Beijing, China).

### 2.4. Animals

Thirty healthy BALB/c male mice (weight: 16–20 g) of specific-pathogen-free (SPF) grade were purchased from Wu’s Laboratory Animal Co., Ltd. The mice were maintained in an environment with air circulation, a temperature of 21–25 °C, and relative humidity of 45–55% [35]. Free food and water were provided. After 1 week of adaptation, the mice were divided into five groups: normal control (NC) (distilled water), low dose (LD) (HGP, 500 mg/kg), middle dose (MD) (HGP, 1000 mg/kg), high dose (HD) (HGP, 2000 mg/kg), and positive control (PC) (octacosanol of 10 mg/kg). The dose of gavage was 0.02 mL/g according to the body weight of the tested animals. Animals were gavaged with HGP at the same time every morning for 6 weeks. The mice were weighed weekly, and the organs were weighed after dissection. The organ index was expressed as organ weight divided by body weight.

### 2.5. Behavioral Tests of Mice

Behavioral tests, comprising weight-loaded swimming test, pole climbing test, passive avoidance test, and elevated cross maze test, were used to evaluate the anti-fatigue ability of the mice.

#### 2.5.1. Weight-Loaded Swimming Test

The weight-loaded swimming test was carried out as described previously [36]. After 30 min of gavage, the mice were placed in a swimming pool (50 × 50 × 40 cm) with water temperature of 25 ± 1 °C and water depth of 30 cm to make sure that their feet could not touch the bottom support. Each mouse had a lead sheet attached to its tail root, weighing 5% of its body weight [37]. When the mouse sank into the water and could not breathe out of the water for 5 s, it was regarded as exhausted. The time from start to exhaustion was recorded by a stopwatch.

#### 2.5.2. Pole Climbing Test

The pole climbing test is used to test the locomotor ability of animals and is a commonly used method to evaluate the fatigue resistance function. The pole climbing test was conducted as described previously [38]. The height and diameter of the pole were 50 cm and 1 cm, respectively. The top of the pole was equipped with a small ball with a diameter of 2.5 cm. To ensure that the surface of the pole was rough, the small ball and the pole were wrapped with adhesive tape. After the mice were given HGP for 30 min, they were placed head-up on the top ball of the pole, and the muscles of the mice were in a tense state at that time. When they reached a state of fatigue, the mice turned their heads and slid off the pole. The time to turn around and climb down was recorded. The mice were regularly trained for pole climbing before the experiment. During the experiment, three consecutive measurements were taken to obtain the average value.

#### 2.5.3. Passive Avoidance Test

The laboratory was kept quiet and ventilated, with a constant temperature of 25 °C and humidity of 55–65%. After 30 min of HGP gavage, the mice were placed in the shuttle box device and subjected to a total duration of 480 s of the passive avoidance test [39]. The test parameters were set to 20 dB sound cue for 4 s, 0.5 mA electric shock stimulation for 5 s after the sound cue, and 6 s for each cycle interval. The time when the mouse showed no shuttle behavior was the lurking time. If the mouse escaped to a safe area during the sound prompt, it was called active escape. If the mouse escaped to the safe area during the electric shock stimulation, it was regarded as passive escape. If the mouse did not escape to the safe area after receiving electrical stimulation, it was regarded as one error. The lurking time, number of shuttle trips, active escapes, passive escapes, and errors were recorded.

#### 2.5.4. Elevated Cross Maze Test

The laboratory was kept quiet and ventilated at a constant temperature of 25 °C and constant humidity of 50–60%. The maze consisted of two open arms (50 × 10 cm), two closed arms (50 × 10 × 40 cm), and a central platform (10 × 10 cm) connecting the four arms. The open and closed arms were perpendicular to each other in a “+” shape, and there was a 1 cm high baffle around the edge of the open arm to prevent animals from sliding down the maze. Before the test, the animals entered the test room 1 h in advance to adapt to the environment. During the test, the fixed experimenter gently grasped the mouse and quickly placed it on the central platform. When placing the mouse, the head was facing one of the open arms. The camera started after the release. The test time for each mouse was 5 min. The video system recorded the behavior of the mice, which was observed through the monitor. The feces of each mouse were removed after testing so as not to affect the behavior of the next mouse. After all the tests were finished, the total distance moved by the mice was recorded and calculated, as well as the distance and percentage of movement in the open arms [40].

### 2.6. Determination of Biochemical Indexes Related to Anti-Fatigue Activity

After gavage of HGP for 30 min, the mice in each group were placed in swimming equipment for 30 min, and orbital blood was collected after 30 min of rest. Serum was prepared by centrifugation (4 °C, 1007× *g*, 10 min) for subsequent experiments. After the mice were dissected, the liver and brain were collected to determine the relevant biochemical indexes. According to the Solarbio kit instructions, lactate dehydrogenase (LDH), blood lactic acid (BLA), blood urea nitrogen (BUN), creatine kinase (CK), liver glycogen (LG), and muscle glycogen (MG) were measured respectively. Malondialdehyde (MDA), catalase (CAT), glutathione peroxidase (GPX), and superoxide dismutase (SOD) in serum and brain tissue were also measured [16].

### 2.7. Statistical Analysis

All experiments were carried out at least three times, and the results are expressed as mean ± standard deviation. SMART v3.0 was used to record and analyze behavioral experimental data. SPSS 18.0 software was used to analyze the significance of differences between samples (*p* < 0.05), and Origin 9.0 was used to draw graphs. All data were analyzed by Student’s t test, and the presuppositions for these tests were properly validated.

## 3. Results

### 3.1. Antioxidant Activity of HGP In Vitro

**Figure 1:**
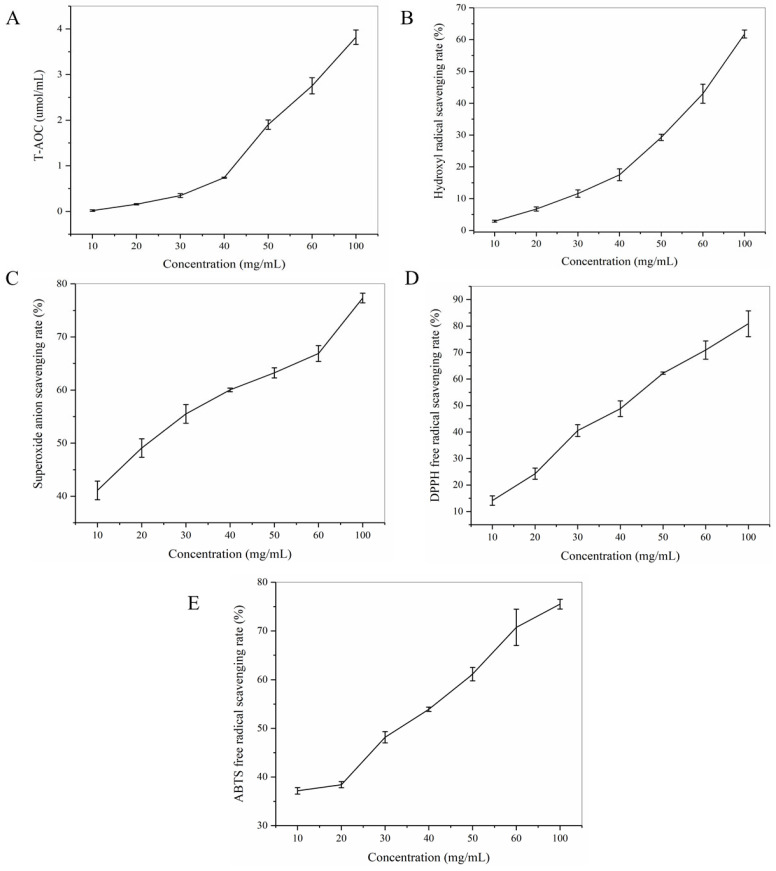
Antioxidant activity of HGP in vitro. (**A**) Total antioxidant capacity (T-AOC) of HGP. (**B**) Hydroxyl radical scavenging rate of HGP. (**C**) Superoxide anion scavenging rate of HGP. (**D**) DPPH radical scavenging rate of HGP. (**E**) ABTS radical scavenging rate of HGP. Results expressed as mean ± standard deviation (*n* = 3).

There is a close correlation between anti-fatigue activity and antioxidant activity. Excessive accumulation of free radicals can lead to fatigue, and the mechanism of antioxidant activity is closely related to scavenging free radicals [41,42]. By drawing a standard curve, the linear regression equation of the total antioxidant capacity was obtained as y = 16.387× + 0.2484 (R^2^ = 0.9995), and the results of the total antioxidant capacity of HGP are shown in Figure 1A. The antioxidant capacity of HGP increased gradually with concentrations from 10 to 100 mg/mL. When the concentration was 60 mg/mL, the total antioxidant capacity (Figure 1A), hydroxyl radical scavenging capacity (Figure 1B), superoxide anion scavenging capacity (Figure 1C), DPPH radical scavenging capacity (Figure 1D), and ABTS radical scavenging capacity (Figure 1E) of HGP reached 2.75 ± 0.18 µmol/mL, 43.0 ± 2.97%, 66.88 ± 1.48%, 70.95 ± 3.42%, and 70.73 ± 3.73%, respectively. When the concentration of HGP was increased to 100 mg/mL, the above indices were 3.82 ± 0.16 µmol/mL, 61.77 ± 1.26%, 77.33 ± 0.91%, 80.88 ± 4.86%, and 75.50 ± 1.00%, respectively, and the above indices were 51.55%, 96.08%, 78.28%, 87.62%, and 89.39% of vitamin C (1 mg/mL), respectively (Table 1).

### 3.2. Effects on Behavior of Experimental Mice

The direct or indirect indexes to evaluate anti-fatigue activity included weight-loaded swimming test, pole climbing test, passive avoidance test, and elevated cross maze test.

#### 3.2.1. Weight-Loaded Swimming Test and Pole Climbing Test

**Figure 2:**
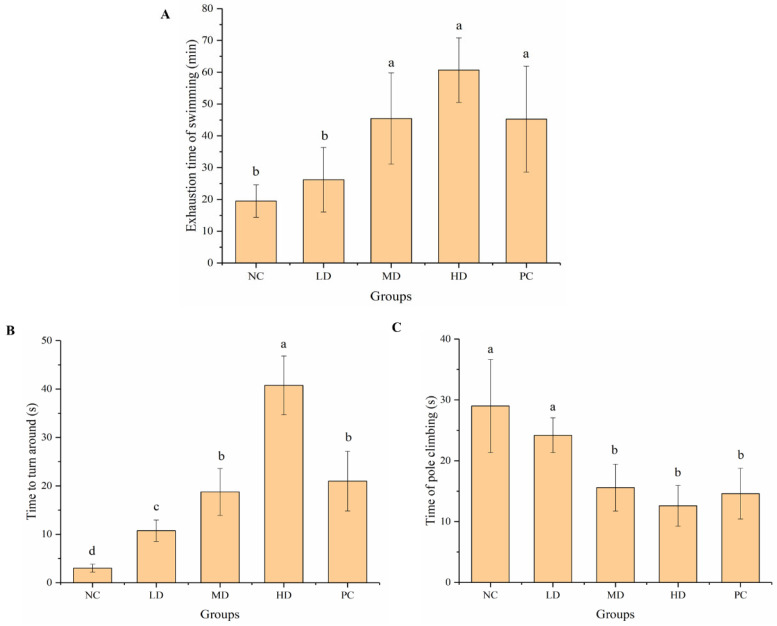
Results of weight-loaded swimming and pole climbing tests. (**A**) Exhaustion time of swimming in mice from different groups. (**B**) Time to turn around in mice from different groups. (**C**) Time of pole climbing in mice from different groups. Different lowercase letters represent significant differences between groups (*p* < 0.05). NC: normal control, LD: low dose, MD: middle dose, HD: high dose, PC: positive control.

The weight-loaded swimming test is a classic behavioral test that reflects exercise ability and is widely used to evaluate anti-fatigue activity. The exhaustion time of mice in the NC, LD, MD, HD, and PC groups was 19.52 ± 5.11, 26.21 ± 10.15, 45.42 ± 14.34, 60.67 ± 10.16, and 45.27 ± 16.67 min, respectively (Figure 2A). The exhaustion time of mice in the LD, MD, and HD groups was higher than that in the NC group, and significantly (*p* < 0.05) different in the MD and HD groups. The exhaustion time of mice in the MD and HD groups was higher than that in the PC group. The results showed that HGP, especially in the HD group, significantly (*p* < 0.05) improved the exercise tolerance of mice.

The time to turn around of mice in the NC, LD, MD, HD, and PC groups was 3.0 ± 0.82, 10.75 ± 2.22, 18.75 ± 4.86, 40.75 ± 6.08, and 21.0 ± 6.16 min, respectively (Figure 2B). The time that mice overcame their body weight and stayed at the top was prolonged in the HGP groups, especially in the HD group. The anti-fatigue ability of mice in the HD group was significantly (*p* < 0.05) stronger than that in the PC group. The time of pole climbing in the NC, LD, MD, HD, and PC groups was 29.0 ± 7.65, 24.20 ± 2.86, 15.60 ± 3.85, 12.60 ± 3.36, and 14.60 ± 4.16 min, respectively (Figure 2C). The mice in the MD and HD groups performed better than those in the NC group when climbing from top to bottom, which was similar to the PC group. Feeding medium and high doses of HGP significantly (*p* < 0.05) improved the mobility of the mice, and the effect was consistent with that in the PC group.

#### 3.2.2. Passive Avoidance Test

**Figure 3:**
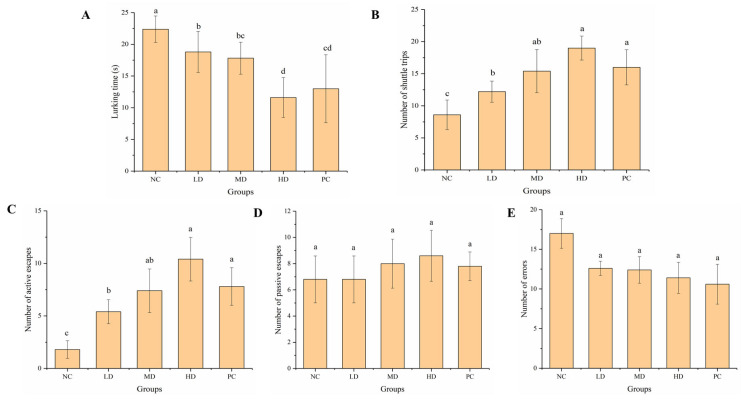
Results of passive avoidance test. (**A**) Lurking time in mice from different groups. (**B**) Number of shuttle trips in mice from different groups. (**C**) Number of active escapes in mice from different groups. (**D**) Number of passive escapes in mice from different groups. (**E**) Number of errors in mice from different groups. Different lowercase letters represent significant differences between groups (*p* < 0.05). NC: normal control, LD: low dose, MD: middle dose, HD: high dose, PC: positive control.

The passive avoidance test can reflect changes in the movement and memory of experimental animals [43,44]. We measured the lurking time, number of shuttle trips, active escapes, passive escapes, and errors (Figure 3). HGP improved the exercise ability of the mice (Figure 3A,B), and the mice in the HD group had the strongest exercise ability. After treatment with HGP, the mice had better conditioned reflexes to the unconditioned stimulus (electric shock) in the shuttle box, suggesting improved learning ability, which indirectly reflected an improved mental state (Figure 3C–E). The passive avoidance test showed that HGP improved the exercise ability of the mice, and fatigue state was relieved.

#### 3.2.3. Elevated Cross Maze Test

**Figure 4:**
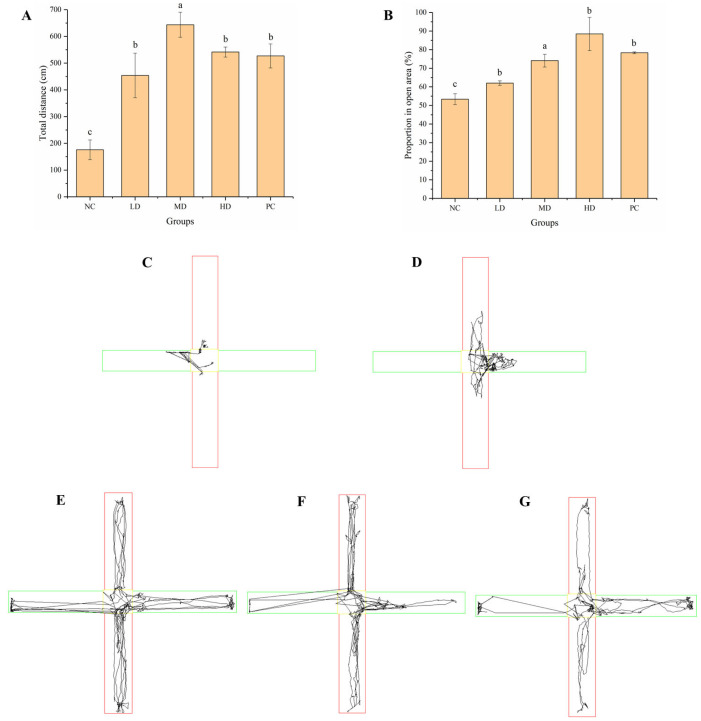
Results of elevated cross maze test. (**A**) Total exercise distance of mice from different groups. (**B**) Proportion of time in open area of mice from different groups. (**C**–**G**) Movement trajectories of mice from different groups. The open area is marked in green lines, the red lines represents the closed area, and the central area is marked in yellow lines. Different lowercase letters represent significant differences between groups (*p* < 0.05). NC: normal control, LD: low dose, MD: middle dose, HD: high dose, PC: positive control.

The elevated cross maze test is a test to reflect the activity of the animals. The increase in time in the open area reflected the increase in the degree of animal activity. The total exercise distance in the MD group was the longest, and the length of time in the open area was longest in the HD group (Figure 4A,B). HGP improved the activity of the mice, indicating that HGP relieved fatigue. The movement trajectories of the mice in each group more intuitively indicated their activity. The exercise capacity and mental activity of mice in the MD and HD groups were significantly (*p* < 0.05) enhanced when compared with the NC group, which was similar to that in the PC group (Figure 4C–G).

### 3.3. General Health and Body and Organ Weights

All the animals looked lively with smooth hair and healthy fecal pellets. No mice experienced diarrhea or constipation. The growth of the mice was normal during the experimental period of 6 weeks without abnormal diseases or deaths. Body weight changes in mice during gavage can reflect the effect of HGP on the appetite or health status of mice [17]. There was no significant difference in the body weight of the mice in each group during gavage, and there was no significant difference between the HGP intervention and PC groups, indicating that HGP had no significant effect on the appetite or health status of the mice (Table 2). Despite the same feeding conditions, the obvious weight gain in the first week in the NC group may have been due to the adaptation period of the mice (Table 2). The tissue weights of the different groups were compared at the end of the experiment. The organ index directly reflects the growth performance of mice and is a common physiological index in animal experiments, which is expressed as organ weight divided by body weight. The gastrocnemius index was significantly increased in the HD group, indicating that HGP improved the muscle movement of the mice (Table 3). There was no significant effect on brain tissue and liver weight.

### 3.4. Effect of HGP on Serum Biochemical Indexes

**Figure 5:**
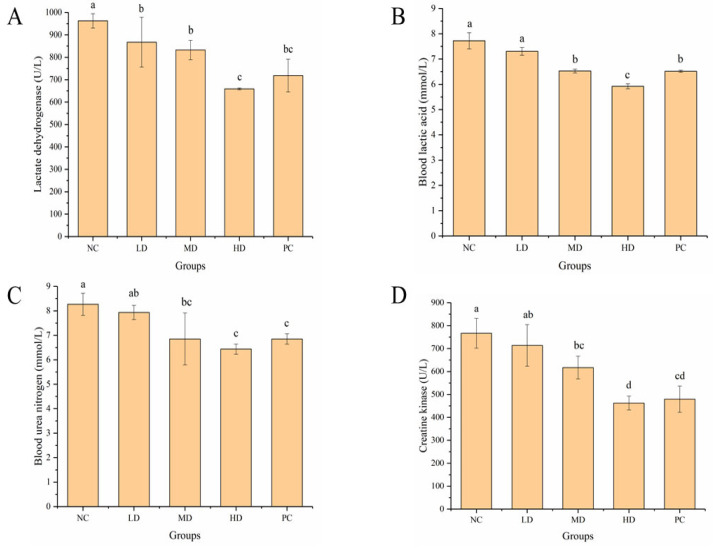
Effect of Hairtail fish (*Trichiurus lepturus*) glycoprotein (HGP) on serum biochemical indexes in mice. (**A**) Lactate dehydrogenase (LDH) in serum of mice from different groups. (**B**) Blood lactic acid (BLA) in serum of mice from different groups. (**C**) Blood urea nitrogen (BUN) in serum of mice from different groups. (**D**) Creatine kinase (CK) in serum of mice from different groups. Different lowercase letters represent significant differences between groups (*p* < 0.05). NC: normal control, LD: low dose, MD: middle dose, HD: high dose, PC: positive control.

During strenuous exercise, the body produces pyruvate under the action of LDH, resulting in BLA accumulation. LDH is an important enzyme in the glycolytic pathway and is often used as an indicator for the evaluation of muscle damage. Compared with the NC group, the level of LDH in serum was significantly (*p* < 0.05) lower in the LD, MD, and HD groups and was the lowest in the HD group, which was similar to the PC group (Figure 5A). HGP blocked the production of BLA and reduced muscle damage caused by excessive accumulation of BLA. BLA is a metabolic product of anaerobic respiration, and the more it accumulates in the body, the more seriously it affects the internal environment, and the degree of fatigue deepens. BLA levels in the LD, MD, and HD groups were 7.30 ± 0.15, 6.53 ± 0.08, and 5.92 ± 0.10 mmol/L, respectively, and BLA in the HD and MD groups was significantly (*p* < 0.05) lower than in the NC group at 7.72 ± 0.32 mmol/L (Figure 5B). The BLA level in the HD group (5.92 ± 0.10 mmol/L) was significantly (*p* < 0.05) lower than in the PC group (6.52 ± 0.04 mmol/L). This suggested that the gavage of HGP promoted the removal of BLA, thus relieving fatigue.

Under normal physiological conditions, the production and elimination of BUN is in balance and remains stable. However, during prolonged vigorous exercise, the energy balance in the body is disrupted and BUN is increased. BUN levels in the LD, MD, and HD groups were 7.93 ± 0.29, 6.85 ± 1.06, and 6.43 ± 0.21 mmol/L, respectively, which were lower than in the NC group at 8.27 ± 0.45 mmol/L (Figure 5C). There were significant (*p* < 0.05) differences in the MD and HD groups compared with the NC group. The effect of reducing the BUN level in the MD and HD groups was similar to that in the PC group (6.85 ± 0.21 mmol/L). The results showed that HGP slowed the metabolism of proteins and amino acids and reduced BUN, thus exerting an anti-fatigue effect. CK is often used as an important biomarker of muscle injury after heavy exercise. The concentration of CK in serum is usually low under normal conditions. However, strenuous exercise can lead to muscle damage and increase cell membrane permeability, and then CK in muscle cells is released into the blood. Compared with the NC group, the CK content in the MD and HD groups was significantly (*p* < 0.05) reduced and was similar to that in the PC group (Figure 5D). This indicated that HGP reduced muscle damage in the mice and thus delayed fatigue.

### 3.5. Effect of HGP on LG and MG

**Figure 6:**
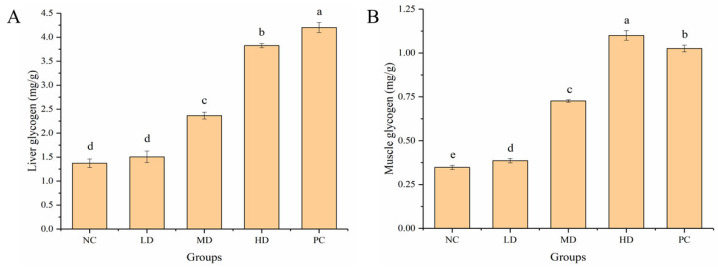
Effect of HGP on liver and muscle glycogen. (**A**) Liver glycogen (LG) of mice from different groups. (**B**) Muscle glycogen (MG) of mice from different groups. Different lowercase letters represent significant differences between groups (*p* < 0.05). NC: normal control, LD: low dose, MD: middle dose, HD: high dose, PC: positive control.

Glycogen is mainly found in the liver and muscle tissue. After a long period of exercise, the body consumes a large amount of glycogen, resulting in fatigue. The LG content of mice in the MD and HD groups was 1.72 and 2.79 times that of mice in the NC group, respectively (Figure 6A). The content of MG in the HGP groups was significantly (*p* < 0.05) higher than that in the NC group, and the MG content in the MD and HD groups was 2.09 and 3.16 times that of mice in the NC group, respectively (Figure 6B). The results showed that HGP delayed the onset of fatigue by reducing the consumption of LG and MG in the mice.

### 3.6. Effect of HGP on Serum and Brain Antioxidant Levels

**Figure 7:**
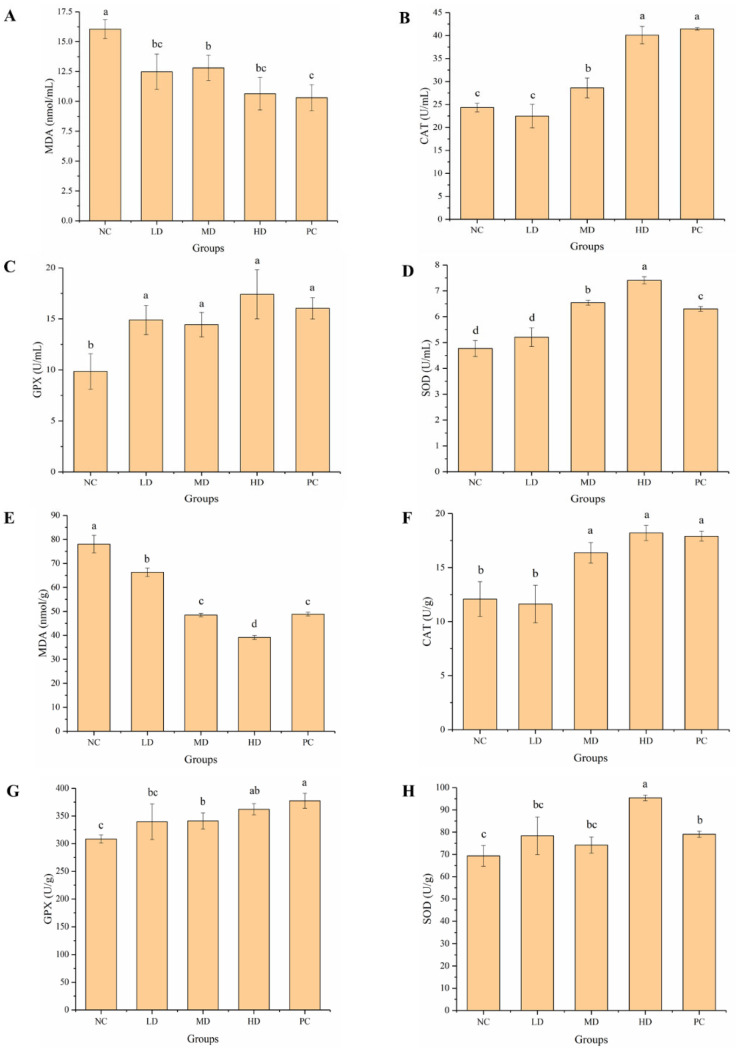
Effect of HGP on serum and brain antioxidant levels in mice. (**A**) Malondialdehyde (MDA) in serum of mice from different groups. (**B**) Catalase (CAT) in serum of mice from different groups. (**C**) Glutathione peroxidase (GPX) in serum of mice from different groups. (**D**) Superoxide dismutase (SOD) in serum of mice from different groups. (**E**) MDA in brains of mice from different groups. (**F**) CAT in brains of mice from different groups. (**G**) GPX in brains of mice from different groups. (**H**) SOD in brains of mice from different groups. Different lowercase letters represent significant differences between groups (*p* < 0.05). NC: normal control, LD: low dose, MD: middle dose, HD: high dose, PC: positive control.

The antioxidant enzymatic systems of the body include MDA, CAT, GPX, and SOD. Oxidative stress plays an important role in the occurrence of fatigue. Improvement of the antioxidant capacity helps to repair damage to cells and mitochondria caused by free radicals generated during exercise, thus delaying fatigue. MDA content in the serum of mice in the LD, MD, and HD groups was significantly lower (*p* < 0.05) than that in the NC group, and MDA content in the serum of mice in the HD group was similar to that in the PC group (Figure 7A). Compared with the NC group, the activities of CAT (Figure 7B), GPX (Figure 7C), and SOD (Figure 7D) in the serum of mice in the MD and HD groups were significantly (*p* < 0.05) increased, and SOD in the serum of mice in the HD group was significantly (*p* < 0.05) higher than that in the PC group. The effect of HGP on oxidative stress in mouse brain was further investigated (Figure 7E–H). Compared with the NC group, the content of MDA (Figure 7E) in the LD, MD, and HD groups was significantly (*p* < 0.05) decreased by 15.02%, 37.89%, and 49.83%, respectively. The activities of CAT (Figure 7F), GPX (Figure 7G), and SOD (Figure 7H) in the brains of the MD and HD groups were significantly (*p* < 0.05) increased, and SOD in the HD group was significantly (*p* < 0.05) higher than that in the PC group. Compared with the PC group, the mice in the HD group showed significantly higher (*p* < 0.05) antioxidant activity. These results suggest that HGP can delay fatigue by repairing cell damage caused by exercise fatigue and increasing antioxidant capacity.

## 4. Discussion

In recent years, studies have found that excessive production of free radicals is an important cause of fatigue. Long-term work and long-term and high-intensity exercise can lead to an excessive increase in free radicals, which can easily lead to fatigue. The mechanism of antioxidation is closely related to the scavenging of free radicals, and a series of studies on the relationship between anti-fatigue activity and antioxidant activity have been carried out. Studies have shown that anti-fatigue function is closely related to antioxidant activity. Wang et al. [42] found that mackerel peptide had good free radical scavenging ability and also prolonged the swimming time of mice, indicating that anti-fatigue is closely related to antioxidant activity. Therefore, the anti-fatigue effect of HGP was evaluated through in vitro antioxidant and in vivo animal experiments in this study. Excessive production of free radicals is an important cause of fatigue, and the improvement of antioxidant capacity can help eliminate free radicals generated in exercise, thereby delaying fatigue [45]. The in vitro antioxidant study found that HGP had good free radical scavenging ability, and the antioxidant activity increased with the concentration. When the concentration reached 100 mg/mL, the effect was similar to that of vitamin C (1 mg/mL), indicating that HGP had good anti-fatigue potential. These results can lay the foundation for follow-up research on the anti-fatigue effect of HGP in BALB/c mice.

Fatigue can be divided into exercise and mental fatigue. Exercise-induced fatigue means that the body cannot maintain a certain intensity of exercise. The weight-loaded swimming test can be used to reflect the exercise endurance of animals, which is the most commonly used model to evaluate anti-fatigue effect. The weight-loaded swimming time can directly reflect the level of exercise endurance, which is important for evaluating the exercise endurance and anti-fatigue effect [46,47]. Ren et al. [48] found that mice treated with grass carp polypeptide had a higher exhaustive swimming time than the control group. Yang et al. [37] found that macamides can improve the exercise tolerance of mice in weight-loaded swimming tests. The weight-loaded swimming time of mice after gavage of walnut oligopeptides was also found to increase [49]. Wang et al. [42] found that mackerel peptide prolonged the exhaustive swimming time of mice. In our study, the HGP groups, especially HD, showed a significantly improved weight-loaded swimming time, indicating the anti-fatigue effect of HGP. The pole climbing test is another commonly used method to evaluate the anti-fatigue function. The mice were placed head-up at the top of the pole, and when they reached a state of fatigue, they slid off the pole. The time to turn around and the time of pole climbing were measured and recorded. In this study, the mice in the MD and HD groups performed better than those in the NC group when climbing from top to bottom, which was similar to the PC group. Mental fatigue is often caused by a lack of rest or by mental tension. Most people, including adolescents and older people, suffer from mental fatigue. The passive avoidance test and elevated cross maze test can be used to reflect changes in the learning ability, mental state, and activity of animals [50]. In this study, compared with the NC group, the mental state and the learning ability of mice were better in the HGP groups, indicating that HGP inhibited mental fatigue and improved the mental state of mice. The behavioral results showed that HGP improved exercise and mental fatigue in BALB/c mice.

The degree of fatigue of mice can be evaluated by certain biochemical indicators after swimming. LDH is an important enzyme in the glycolysis pathway and is often used as an indicator of muscle damage. BLA is an anaerobic respiratory metabolite. During strenuous exercise, BLA and BUN increase, which seriously aggravates the degree of fatigue [51]. After strenuous exercise, protein metabolism is enhanced and BUN increases, which can be used as an important indicator for evaluating fatigue. CK is often used as an important biomarker of muscle damage after strenuous exercise. Mackerel peptide was found to have anti-fatigue activity by significantly reducing the levels of BUN and BLA [42]. In this study, HGP reduced the levels of metabolites in the serum of mice after strenuous exercise. It can be speculated that HGP exerts anti-fatigue effects by reducing LDH content, blocking BLA production, and reducing BUN and CK levels. In exercise, glycogen is an important source of energy. Most energy is provided by glycogen during exercise [52], and the body experiences fatigue when glycogen is consumed [53]. Glycogen in the liver can be decomposed to provide glucose throughout the body. Mackerel peptide was also found to exert anti-fatigue effects by increasing LG levels in mice [42]. In this study, the LG and MG content of mice increased in the HGP groups, indicating that HGP improved the supply of energy in mice during exercise and delayed the onset of fatigue.

Oxidative stress plays an important role in the occurrence of fatigue. Exercise can lead to excessive production of free radicals and disorder of cell metabolism, which leads to fatigue. Lipid peroxidation is an important mechanism of cell damage mediated by free radicals. It can directly damage the cell membrane, which is considered to be related to various disease processes [54]. MDA is a lipid peroxide produced when the body is attacked by oxygen free radicals, which indirectly reflects the degree of cell damage [55]. In this study, compared with the NC group, the content of MDA in the serum and brain of mice decreased after HGP intervention. CAT, GPX, and SOD are important antioxidant enzymes which can inhibit the production of oxygen free radicals, balance human metabolism, and prevent the occurrence of some diseases. The polysaccharides from *Rollinia parviflora* were found to exert anti-fatigue activity by reducing oxidative stress in mice during strenuous exercise [17]. Ren et al. [48] found that the activities of GPX, SOD, and CAT were all significantly higher in the grass carp polypeptide groups than in the control group, indicating that the grass carp polypeptide had anti-fatigue activity. We also found that HGP improved the activity of CAT, GPX, and SOD in the serum and brain of mice. HGP was an effective antioxidant to inhibit lipid peroxidation and exert anti-fatigue activity by improving oxidative stress and lipid peroxidation in mice.

## 5. Conclusions

We performed in vitro antioxidant experiments, and the anti-fatigue activity of HGP was further evaluated in animal models. The results of the in vitro experiments showed that HGP had good free radical scavenging ability, indicating that HGP has anti-fatigue potential. The results of animal behavioral experiments showed that HGP improved the physical strength of mice and their memory and activity. HGP also reduced the levels of metabolites such as LDH, BLA, BUN, and CK in serum after exercise. The contents of liver and muscle glycogen were also significantly increased by HGP. After HGP treatment, the content of MDA in the serum and brain tissues of the mice was significantly reduced, and the content of antioxidant enzymes such as CAT, GPX, and SOD was significantly increased, especially in the MD and HD groups. These results indicate that HGP can be used as a natural food additive to relieve fatigue and enhance exercise endurance, and they provide a theoretical basis for the application of HGP in the field of functional foods.

## Figures and Tables

**Table 1:** Antioxidant activity of vitamin C in vitro.

T-AOC (µmol/mL)	Hydroxyl Radical Scavenging Rate (%)	Superoxide Anion Scavenging Rate (%)	DPPH Free Radical Scavenging Rate (%)	ABTS Free Radical Scavenging Rate (%)
7.41 ± 0.03	64.29 ± 3.72	98.79 ± 0.26	92.31 ± 0.63	84.46 ± 1.07

Results expressed as mean ± standard deviation (*n* = 3).

**Table 2:** Effect of Hairtail fish (*Trichiurus lepturus*) glycoprotein (HGP) on body weight of experimental mice.

Group	0 Weeks	1 Week	2 Weeks	3 Weeks	4 Weeks	5 Weeks	6 Weeks	7 Weeks
	Weight (g)							
NC	20.78 ± 0.80 ^a^	23.44 ± 1.03 ^a^	24.93 ± 1.20 ^a^	26.40 ± 1.55 ^a^	27.16 ± 1.49 ^a^	28.53 ± 1.50 ^a^	29.34 ± 1.44 ^a^	29.42 ± 1.71 ^a^
LD	20.61 ± 1.03 ^a^	21.77 ± 1.18 ^c^	22.97 ± 1.29 ^b^	24.58 ± 1.24 ^b^	25.91 ± 1.27 ^b^	26.92 ± 1.28 ^b^	27.90 ± 1.23 ^b^	28.09 ± 1.28 ^b^
MD	20.29 ± 0.78 ^a^	22.20 ± 1.07 ^bc^	23.15 ± 0.97 ^b^	24.44 ± 1.07 ^b^	25.58 ± 1.30 ^b^	27.07 ± 1.24 ^b^	27.80 ± 1.14 ^b^	28.16 ± 1.25 ^b^
HD	20.53 ± 1.00 ^a^	22.40 ± 0.92 ^bc^	23.13 ± 1.20 ^b^	24.66 ± 1.54 ^b^	25.73 ± 1.93 ^b^	27.25 ± 1.71 ^b^	28.19 ± 1.70 ^ab^	28.36 ± 1.75 ^ab^
PC	20.57 ± 0.92 ^a^	22.91 ± 1.21 ^ab^	23.26 ± 1.30 ^b^	24.54 ± 1.28 ^b^	25.88 ± 1.37 ^b^	27.42 ± 1.06 ^b^	27.91 ± 1.17 ^b^	28.26 ± 1.14 ^b^

Results are expressed as mean ± standard deviation (*n* = 3). Different lowercase letters represent significant differences between groups (*p* < 0.05). NC: normal control, LD: low dose, MD: middle dose, HD: high dose, PC: positive control.

**Table 3:** Effect of HGP on organ index of experimental mice.

Group	Brain Index (%)	Liver Index (%)	Gastrocnemius Index (%)
NC	1.32 ± 0.09 ^a^	4.72 ± 0.21 ^a^	1.12 ± 0.03 ^b^
LD	1.33 ± 0.11 ^a^	4.75 ± 0.24 ^a^	1.12 ± 0.08 ^ab^
MD	1.36 ± 0.10 ^a^	4.76 ± 0.18 ^a^	1.14 ± 0.06 ^ab^
HD	1.36 ± 0.12 ^a^	4.80 ± 0.18 ^a^	1.17 ± 0.08 ^a^
PC	1.34 ± 0.09 ^a^	4.71 ± 0.25 ^a^	1.14 ± 0.11 ^ab^

Results are expressed as mean ± standard deviation (*n* = 3). Different lowercase letters represent significant differences between groups (*p* < 0.05). NC: normal control, LD: low dose, MD: middle dose, HD: high dose, PC: positive control.

## Data Availability

The data presented in this study are available in Supplementary Materials.

## Supplementary Materials

The following supporting information can be downloaded at: https://www.mdpi.com/article/10.3390/foods12061245/s1, Table S1: Antioxidant Activity of HGP and Vitamin C; Table S2: Behavioral Tests of Mice; Table S3: Body and Organ Weights; Table S4: Biochemical Indexes Related to Anti-Fatigue Activity.

## Abbreviations

HGP	Hairtail fish (*Trichiurus lepturus*) glycoprotein
SOD	Superoxide dismutase
CAT	Catalase
GPX	Glutathione peroxidase
MDA	Malonaldehyde
LDH	Lactate dehydrogenase
BLA	Blood lactic acid
BUN	Blood urea nitrogen
CK	Creatine kinase
LG	Liver glycogen
MG	Muscle glycogen
